# Denosumab Therapy in the Management of Aneurysmal Bone Cysts: A Comprehensive Literature Review

**DOI:** 10.7759/cureus.3989

**Published:** 2019-01-31

**Authors:** Ibrahim Alhumaid, Ahmed Abu-Zaid

**Affiliations:** 1 Orthopaedics, Alfaisal University College of Medicine, Riyadh, SAU; 2 Oncology, Alfaisal University College of Medicine, Riyadh, SAU

**Keywords:** denosumab, aneurysmal bone cyst, management, rank ligand

## Abstract

Aneurysmal bone cysts (ABCs) are uncommon lesions that involve the axial and appendicular bones. Although biologically benign, ABCs have the tendency to assume an aggressive behavior causing local destruction of the underlying bone and neighboring soft tissues. Morphologically, ABCs are composed of cyst-like cavities filled with blood and bounded by an array of diverse cells including fibroblasts, inflammatory infiltrates and osteoclast-like multinucleated giant cells. From a molecular perspective, the osteoclast-like multinucleated giant cells harbor high expression of receptor activator of nuclear factor kappa B (RANK) receptors, whereas the neoplastic stromal cells harbor high expression of RANK ligand (RANKL). The RANK-RANKL interaction has been implicated in the carcinogenesis of ABCs and giant cell tumor of bones (GCTBs). Currently, the optimal management of ABCs remains a hotly debated topic. There are a multitude of treatment modalities (that is, surgery, sclerotherapy, radiotherapy and selective arterial embolization), and each modality has its own benefits, morbidity and risk of complications. The local aggressiveness of ABC and its high rates of relapse following treatment has demanded the march towards discovering more innovative therapies. One of such therapies is denosumab, a monoclonal antibody targeted against the RANKL. Denosumab is already approved by the United States Food and Drug Administration (FDA) for the treatment of adults and skeletally mature adolescents with GCTB that is unamenable to surgery, or initial surgery is anticipated to result in significant morbidity. However, denosumab is not approved by the FDA for the management of ABCs. However, taking into consideration the morphological similarity between GCTBs and ABCs, some treating physicians have logically opted to use denosumab in an off-label manner to treat select ABCs. To the best of knowledge, no study has attempted to summarize the related literature on the use of denosumab in ABCs. Therefore, the primary aim of this study is to narratively review all the available literature about the efficacy and safety of the use of off-label denosumab in the management of patients with ABCs.

## Introduction and background

Aneurysmal bone cysts (ABCs) are infrequent, biologically benign and locally destructive lesions that most commonly take place during the first two decades of life [1]. Clinically, patients with ABCs typically present with pain, swelling, budding mass, bone demolition and sometimes pathological fracture of the underlying bone [2]. ABCs can present as primary or secondary lesions [1-3]. Primary ABCs account for roughly two-thirds (70%) of all cases. Conversely, secondary ABCs account for nearly one-third (30%) of the cases and most often are associated with a wide-ranging spectrum of bone disorders, such as giant cell tumor of bone (GCTB), osteoblastoma, low-grade osteosarcoma and fibrous dysplasia. The most frequent sites of involvement in ABCs comprise the spine (vertebral bodies) and long bones (specifically the metaphysis of the distal femur and proximal tibia), although virtually any bone of the body can be affected by ABCs [4-5]. Involvement of the spine, particularly, is associated with anatomical challenges and increased hazards of neurological deficits, surgical morbidity and recurrence [6-7].

Conventionally, ABCs were believed to arise from a vascular disturbance, specifically increased venous pressure, resulting in amplified intraosseous pressure and extravasation of cellular and blood contents into cyst-like cavities within the bone. These cavities eventually lead to local distension and destruction of the underlying bone and adjacent tissues [8]. However, more recently, it has been shown that upregulation of a characteristic translocation TRE17/USP6 oncogene is implicated in the pathogenesis of ABCs, by promoting increased matrix metalloproteinase production through activation of the receptor activator of nuclear factor kappa B (NF-κB) signaling pathway [8]. From a histological point of view, ABCs comprise large-sized and cyst-like spaces filled with blood and bounded by fibrous septal connective tissues including fibroblasts, spindle cells, inflammatory infiltrates, multinucleated giant cells, osteoid and scattered calcifications [2,7].

From a molecular point of view, ABCs comprise osteoclast-like multinucleated giant cells that express high levels of receptor activator of nuclear kappa B (RANK) receptors and neoplastic stromal cells that express high levels of RANK ligand (RANKL). The RANK-RANKL interaction activates a signaling cascade that promotes abnormally increased bone resorption, osteolysis and destruction seen in patients with ABCs [9-10]. Several reports demonstrated that GCTBs and ABCs share closely similar histopathological features [7,9-11].

At the present time, optimal management of ABCs continues to be a subject of controversy [2]. There are several treatment modalities, and each has its own benefits, morbidity and risk of complications. Surgical resection (en bloc) or intralesional curettage with or without bone grafting and local adjuvants appear to stand as the standard of care (whenever technically feasible), despite being associated with a high morbidity rate [5]. Other treatment options for ABCs comprise sclerotherapy, radiotherapy and selective arterial embolization (SAE) [2,5]. All of these treatment modalities have specific recommendations as well as associated advantages and disadvantages. Although ABCs are biologically benign lesions, they have a great tendency for local aggressiveness and high rates of relapse following treatment. Thus, management of ABCs continues to be distinctively challenging and there is always a pressed necessity for more innovative therapies [12].

Denosumab, a monoclonal antibody targeted against the RANKL, has been shown to exhibit favorable therapeutic benefits in patients with GCTB [13-19]. In mid-2013, denosumab was approved by the United States Food and Drug Administration (FDA) for the treatment of adults and skeletally mature adolescents with GCTB that is unamenable to surgery, or initial surgery is anticipated to result in significant morbidity [13-14,20]. Denosumab is not yet FDA-approved for the management of ABCs. However, taken into account the histopathological resemblance between GCTBs and ABCs [7,9-10], some authors have opted to treat select patients with off-label denosumab therapy. The studies that have reported the role of off-label denosumab in the management of ABCs are scattered throughout literature and not collectively summarized.

Therefore, the primary aim of this study is to narratively review all the available literature about the efficacy and safety of the use of off-label denosumab in the management of patients with ABCs.

## Review

The PubMed® database was screened from 1 January 1990 until 15 January 2019 using the following keywords: “aneurysmal bone cyst” and “denosumab”. Only English-published studies were retrospectively reviewed. Additional references from published articles were also manually screened for potential inclusion in the study analysis. The study inclusion criteria included (i) patients diagnosed with ABCs, (ii) studies reporting clinical retrospective series or case reports and (iii) studies reporting the use of denosumab therapy either in neoadjuvant or adjuvant settings. For each reviewed study, the following details, upon availability, were retrospectively reviewed including authors, year of publication, country of research, study type, study sample size, clinical efficacy, toxicity profile, denosumab dosing, duration of follow-up and survival outcomes.

In 2013, Lange et al. [12] (case series from Germany) reported the efficacy and safety of denosumab in two patients with ABCs. The first case was an 8-year-old male patient with ABC involving the spine (C5). The patient complained of a four-month history of left-sided pain over the cervico-brachial region in addition to torticollis. The patient underwent emergency decompression procedure with immediate pain relief; however, one month later, the patient complained of progressive pain relapse and motor deficits, and imaging studies showed tumor progression. In view of tumor progression and unsuitability for surgical intervention, the patient was treated with off-label denosumab (70 mg/m² once every four weeks). The patient developed hypocalcemia and his calcium supplementation dose was increased from 500 mg to 1000 mg daily. No major denosumab-related adverse events were reported. At the last date of follow-up (two months following denosumab administration), imaging studies with magnetic resonance imaging (MRI) showed peripheral tumor ossification and no evidence of radiological recurrence. The second case was an 11-year-old male patient with ABC involving the spine (C5). The patient complained of a six-month history of right-sided weakness of his forearm. The patient underwent surgical excision with clinical improvement; however, eight months later, the patient complained of progressive pain relapse without motor deficits, and imaging studies showed tumor enlargement. In view of tumor advancement and unfeasibility for surgical intervention, the patient was treated with off-label denosumab (70 mg/m² once weekly, and then once monthly afterward). Despite the intensive regimen of denosumab administration, no major drug-related side effects were reported. At the last date of follow-up (four months following denosumab administration), imaging studies with MRI showed peripheral tumor ossification and no evidence of radiological recurrence.

In 2014, Pauli et al. [21] (case report from Switzerland) reported a 21-year-old female patient with recurrent and unresectable ABC involving the radius. The patient complained of swelling and shooting pain involving the right forearm. The patient received off-label denosumab therapy (120 mg once monthly) for a duration of four months (a total of four doses). The patient had tumor shrinkage within one month following denosumab therapy. At five months post denosumab administration, radiographic images showed downsizing of the tumor with peripheral calcification which subsequently facilitated complete tumor excision. However, upon histopathological analysis, there was a region that was not tumor-free located at the distal portion of radius. The resected specimens had substantially decreased numbers of the osteoclast-like multinucleated giant cells. At the date of last follow-up (19 months following surgery), imaging studies showed a small mass at the site of the distal osteotomy, and core-needle biopsy demonstrated a histological proof of local relapse. The patient was subsequently started on a treatment protocol with denosumab. No denosumab-related adverse events were reported. The authors concluded that denosumab therapy could be utilized as a potential therapy in patients with ABCs.

In 2014, Pelle et al. [9] (a case report from the United States of Ameria) reported a five-year-old male patient with a huge and locally aggressive ABC involving the sacrum. The patient had a three-month history of symptoms of urinary and bowel incontinence in addition to a 12-month history of lower back pain. The patient received off-label denosumab therapy for 12 months (1.2 mg/kg/dose and increased weekly to a final dose of 1.6 mg/kg/dose given monthly after the first month) as an alternative to surgical intervention. The patient experienced initial pain relief and complete pain resolution with mobilization within two and six weeks, respectively. At three months following denosumab therapy, imaging studies showed tumor downsizing, new bone formation and restoration of the underlying pathological fracture. At the date of last follow-up (12 months post denosumab administration), the patient received 10 doses of denosumab therapy, was clinically stable with complete resolution of lower genitourinary symptoms and had no radiological evidence of recurrence. No denosumab-related adverse events were reported throughout the therapy. A retrospective examination of the patient’s biopsy before denosumab therapy showed high expression of RANKL. The authors concluded that denosumab therapy could offer therapeutic benefits and serve as a reasonable substitute therapy to surgery in a specific population of ABC patients.

In 2015, Skubitz et al. [22] (a case report from the United States of America) reported a 27-year-old male patient with a large ABC involving the sacrum. The patient complained of a progressive backache. The tumor was not amenable to safe surgical resection, and thus the patient received off-label denosumab therapy (120 mg on days 1, 8, 15, 29, and then once every four weeks thereafter). The patient experienced initial pain relief and complete pain resolution within three weeks and two months, respectively. At four months follow-up, computed tomography (CT) scan showed new bone formation. The patient received denosumab therapy for 11 months. At the last date of follow-up (12 months post denosumab administration), the patient was clinically well, radiologically stable and histopathological examination of an incisional biopsy showed new bone ossification and absence of the multinucleated giant cells. The authors concluded that denosumab therapy yielded therapeutic, radiological and histological benefits in ABC, and its beneficial utility could be limited to select cases.

In 2016, Ghermandi et al. [7] (a case report from Italy) reported the efficacy and safety of denosumab in two patients with ABCs. The first case was a 42-year-old male patient with an ABC involving the lumbar spine. The patient had a two-year history of lower back pain. The patient failed several treatment trials with SAE. Considering the record of tumor resistance and potential surgical morbidity, the patient was started on an off-label denosumab therapy (120 mg once weekly for four weeks and then once every six weeks). At two months follow-up, the patient was symptom-free and imaging studies demonstrated significant tumor calcification. At 35 months after clinical diagnosis (the date of the last follow-up in the clinic), the patient had received a sum of 13 doses of denosumab and was symptom-free without any clinical or radiological proof of recurrence. The second case was a 16-year-old male patient with an ABC involving the lumbar spine. The patient had a four-month history of lower back pain as well as pain and weakness involving the right leg. The patient failed several treatment trials with SAE. Considering the history of tumor resistance and probable intraoperative morbidity, the patient was started on an off-label denosumab therapy (120 mg once weekly for four weeks and then once every six weeks). The patient exhibited substantial clinical improvement within one month, and imaging studies displayed substantial calcification and lesion shrinkage by the end of the third month of denosumab administration. At 33 months after clinical diagnosis (the date of the last follow-up in the clinic), the patient had received a sum of 11 doses of denosumab and was symptom-free without any clinical or radiological evidence of recurrence. In both cases, the tumor was extensively ossified. The authors concluded that denosumab therapy was clinically and radiologically effective and could surface as a potential substitute therapy in patients with SAE-resistant ABCs.

In 2016, Dubory et al. [23] (case series from France) reported the efficacy and safety of denosumab in nine patients with GCTB and ABC. Only one patient had the histology of ABC. This patient was a 26-year-old female with a fairly large ABC involving the spine (C7-T1). At the time of clinical presentation, the patient was pregnant and complained of wide-ranging severe neuralgia pertaining to cervicobrachial impingement. Due to sudden paraplegia secondary to spinal cord compression by the tumor, the patient underwent an emergency caesarian section, cervical laminectomy and C4-T4 fixation. Postoperatively, the patient received adjuvant off-label denosumab therapy for a minimum of six months. The exact duration of denosumab therapy was not reported for the ABC patient; however, the duration of denosumab therapy for the entire patient cohort (*n *= 9) ranged from three to 24 months (mean 13 months). Similarly, the exact follow-up period was not reported for the ABC patient, however, the duration of follow-up for the entire patient cohort (*n *= 9) ranged from three to 52 months (mean 19 months). The patient did not have a clinical response as the patient had persistent neurologic symptoms. Radiologically, there was a fairly slight reduction of the lesion size with peripheral ossification. No denosumab-related adverse events were reported. For the entire patient cohort (eight patients with GCTBs and one patient with ABC), the authors concluded that denosumab therapy permitted tumor shrinkage and bone consolidation and that denosumab should not replace definitive surgery. Furthermore, the authors highlighted the need to investigate the potential of local recurrence upon the cessation of denosumab therapy.

In 2017, Ntalos et al. [24] (case report from Germany) reported a 35-year-old female patient with a primary ABC involving the pelvis that was not amenable to surgical intervention. The patient complained of right-sided pain and gluteal swelling. The patient underwent SAE as well as off-label denosumab (60 mg once every four weeks) for a total of 12 months. Within three months, the patient reported substantial pain relief. Moreover, CT scans displayed reduced tumor size, increased tumor ossification and decreased cystic cavity mineralization. Due to downsizing of tumor mass, the patient underwent successive surgery (intralesional curettage, bone grafting and adjuvant cementing) in addition to preoperative SAE. At six months postoperatively, CT scan showed evidence of tumor recurrence in terms of increased tumor size and reduced tumor rim calcification. The patient was re-treated again with denosumab therapy and exhibited favorable clinical and radiological responses. The patient was tolerating the drug until the patient developed significant hypocalcemia necessitating immediate termination of denosumab therapy after a sum of 17 months administration of denosumab. At the last time of follow-up (four years), the patient was clinically and radiologically stable after approximately 16 months of final suspension of denosumab therapy. The authors concluded that denosumab could be regarded as a plausible therapy in the management of patients with ABC.

In 2018, Fontenot et al. [25] (a case report from the United States) reported a 13-year-old female patient with a history of recurrent ABC involving the distal fibula (long bone). The patient had severe pain and limited activity of his forearm. The patient received preoperative denosumab for one year (120 mg given every four weeks with additional 120 mg subcutaneous doses on days 8 and 15 in cycle one) followed by intralesional curettage with high-speed burring and cement augmentation. Postoperative histopathological examination of the open biopsy showed substantial resolution of the neoplastic multinucleated giant cells. At three-year follow-up, the patient had improved quality of life in terms of pain relief and restored the functional activity of forearm. No denosumab-related side effects were reported. Furthermore, there was no clinical or radiological proof of recurrence. The authors concluded that the use of preoperative denosumab offered clinical benefits, reduced local recurrence and exhibited a safe drug profile in a patient with recurrent ABC.

In 2018, Asi et al. [26] (a case report from the United States of America) reported a 32-year-old male patient with a massive primary ABC involving the maxillary sinus and anterior skull base. The patient had significant uncontrolled tumor-related pain and multiple progressive cranial neuropathies. Due to reasons of difficult anatomical location and potential surgery-related morbidity, the patient received preoperative denosumab treatment (120 mg subcutaneous injections weekly for one month, followed by monthly administration). Clinical benefits were observed within one month following denosumab treatment. At roughly 18 months follow-up, the patient had improved tumor-related pain and dramatic resolution of the cranial neuropathies. No denosumab-related side effects were reported. Radiologically, the tumor displayed a substantial reduction in metabolic activity and extensive ossification, despite no downstaging in the size of the tumor. Considering the beneficial response to denosumab therapy, the managing team decided to proceed with monthly denosumab administration; surgery was regarded as the last therapeutic resort. The authors concluded that denosumab therapy was effective clinically and radiologically and offered a reliable substitute for surgery in patients with ABCs involving high-risk anatomical locations.

In 2018, Patel et al. [27] (a case report from Singapore) reported a 16-year-old male patient with a large ABC involving the vertebral arch of atlas (C1). The patient complained of severe neck pain and limited range of motion. The patient received off-label denosumab for 12 months (120 mg monthly). An initial positive response to therapy was noticed at six weeks post denosumab therapy, and symptoms were completely resolved with pain relief and restoration of full range of neck motion. No denosumab-related side effects were reported throughout the treatment course. At 12 months follow-up, CT scans showed the tumor was extensively ossified without any clinical or radiological proof of recurrence. The authors concluded that denosumab therapy was associated with favorable clinical and drug-related outcomes in a patient with spinal ABC.

In 2018, Palmerini et al. [4] (case series from Italy) examined the efficacy and safety of denosumab in nine patients with ABCs involving the spine-pelvis (*n *= 6), humerus (*n *= 1), ulna (*n *= 1) and tibia (*n *= 1). These patients were treated with off-label denosumab owing to either inability to perform surgery or as executing SAE was not practically feasible. The number of denosumab administrations (subcutaneous injection in the dose of 120 mg on days one, eight, 15 and 29 and then once every four weeks) ranged from three to 61 cycles (median eight cycles; mean 21 cycles). The duration of follow-up ranged from three to 55 months (median 23 months). All the symptomatic patients (*n *= 8) had substantial clinical pain relief and improvement. Radiologically, all patients (*n *= 9) had tumor ossification observed by CT, whereas only seven out of the nine patients had a reduction in the MRI gadolinium contrast media uptake. Overall, at the time of the last follow-up, all patients were free of disease progression. Specifically, two patients received preoperative denosumab therapy and underwent successive intralesional curettage surgery after five and nine months of denosumab therapy. Postoperative histopathological examination of specimens showed complete resolution of the neoplastic multinucleated giant cells in both patients. Two patients were progression-free at 12 and 24 months after denosumab cessation and did not undergo surgery. The remaining five patients were still receiving denosumab therapy and progression-free at the time of the last follow-up. Only one patient presented with grade-I vomiting that was successfully treated medically. No major denosumab-related side effects were reported. The authors concluded that denosumab therapy was clinically useful and tolerable in the management of patients with unresectable, locally advanced or recurrent ABCs.

In 2018, Kurucu et al. [28] (case series from Turkey) investigated the efficacy and tolerability of denosumab in nine patients with ABCs involving the pelvis (*n *= 4), vertebrae (*n *= 2), humerus (*n *= 1) and mandible (*n *= 1). Three and six patients had recurrent and primary ABC diseases, respectively. All patients received off-label denosumab therapy (70 mg/m^2 ^was administered weekly in the first month and then once monthly afterward). The number of denosumab doses ranged from nine to 17 doses (median 15 doses), and the duration of denosumab therapy ranged from six to 14 months (median 12 months). The duration of follow-up after the start of denosumab therapy ranged from 24 to 33 months (median 29 months), whereas the duration of follow-up after the completion of denosumab treatment ranged from 10 to 24 months (median 15 months). Clinically, all patients experienced symptom improvement and complete regression within one and three months, respectively. Radiologically, denosumab therapy demonstrated wide-ranging radiological efficacy, in terms of overall ABC volume reduction (*n *= 6), reduced cyst number/size (*n *= 8), decreased MRI-T2 signal (*n *= 8) and reduced fluid–fluid levels in cysts (*n *= 5). Overall, seven patients were clinically and radiologically responsive to denosumab therapy. At the time of the last follow-up, six patients had stable disease, whereas three patients underwent surgical intervention for clinical (*n *= 1) or radiological (*n *= 2) recurrences. Overall, the denosumab therapy was well-tolerated. During treatment, denosumab-related adverse events included mild fatigue (*n *= 2), mild muscle ache (*n *= 1) and mild nausea/vomiting (*n *= 1). Five months following completion of denosumab therapy, two patients developed severe hypercalcemia, who had received 17 doses of denosumab. The authors concluded that the administration of denosumab therapy was associated with favorable clinical, radiological and drug-related tolerability outcomes in patients with surgically challenging and morbid ABCs.

Table 1 shows a summary of all published literature about the use of denosumab in the management of patients with ABCs. Overall, 12 studies were reviewed, with a total of 30 patients with recurrent or metastatic NPC were treated with denosumab. The vast majority of studies were isolated case reports (*n *= 8) originated from United States (*n *= 4) and had patients under 18 years of age (*n *= 20). The bulk of patients had ABCs involving the spine (*n *= 13; cervical, thoracic or lumbar spines) and the sacrum (*n *= 4). Almost all the evaluable patients treated with denosumab experienced clinical (*n *= 27/28), radiological (*n *= 28/30) and histological (*n *= 6/7) responses. The duration of the follow-up differed significantly and ranged from as low as two months to as high as 48 months (rough median is 20 months). With respect to denosumab-related side effects, a sum of eight adverse events was reported as follows: hypocalcemia (*n *= 2), hypercalcemia (*n *= 2), mild vomiting (*n *= 2), mild fatigue (*n *= 1) and mild back pain (*n *= 1). No denosumab-related mortality was reported. At the time of the last follow-up, almost all patients were alive with stable disease (*n *= 26).

**Table 1:** A summary of all published literature about the use of denosumab in the management of patients with aneurysmal bone cysts (ABCs) F: female; M: male; *n*: patient sample size; ref: reference; USA: United States of America; NR: non renseigné (not specified) * The exact duration of follow-up per patient was not stated. However, for the entire patient cohort, the median duration of follow-up was 23 months (range: 3-55 months). ** Overall, the study reported the following denosumab-related adverse events: mild fatigue (*n *= 2), mild muscle ache (*n *= 1) and mild nausea/vomiting (*n *= 1). However, the assignment of each adverse event to the specific patient was not stated. Moreover, after the completion of denosumab therapy, two patients developed severe hypercalcemia (received 17 does); however, the exact both patients were not specified. *** Spine tumor site includes cervical, thoracic or lumbar spines.

Ref	Authors	Year	Country	(n)	Patient age/sex	Tumor site***	Clinical response	Radiological response	Histological response	Complications	Follow-up (months)	Status
[12]	Lange et al	2013	Germany	2	8-year-old/M	Spine	Yes	Yes	NR	Hypocalcemia	2	Stable
					11-year-old/M	Spine	NR	Yes	NR	None	4	Stable
[21]	Pauli et al	2014	Switzerland	1	21-year-old/F	Radius	Yes	Yes	Yes	None	19	Recurrence
[9]	Pelle et al	2014	USA	1	16-year-old/M	Sacrum	Yes	Yes	NR	None	12	Stable
[22]	Skubitz et al	2015	USA	1	27-year-old/M	Sacrum	Yes	Yes	Yes	None	12	Stable
[7]	Ghermandi et al	2016	Italy	2	42-year-old/M	Spine	Yes	Yes	NR	None	35	Stable
					16-year-old/M	Spine	Yes	Yes	NR	None	33	Stable
[23]	Dubory et al	2016	France	1	26-year-old/F	Spine	No	Yes	NR	None	NR	Stable
[24]	Ntalos et al	2017	Germany	1	35-year-old/F	Pelvis	Yes	Yes	NR	Hypocalcemia	48	Stable
[25]	Fontenot et al	2018	USA	1	13-year-old/F	Fibula	Yes	Yes	Yes	None	36	Stable
[26]	Asi et al	2018	USA	1	32-year-old/M	Skull base	Yes	Yes	NR	None	18	Stable
[27]	Patel et al	2018	Singapore	1	16-year-old/M	Spine	Yes	Yes	NR	None	12	Stable
[4]	Palmerini et al	2018	Italy	9	14-year-old/F	Sacrum	None	Yes	NR	None	23*	Stable
					16-year-old/M	Spine	Yes	Yes	NR	None	24	Stable
					42-year-old/M	Spine	Yes	Yes	NR	None	23*	Stable
					16-year-old/F	Pelvis	Yes	Yes	NR	None	23*	Stable
					12-year-old/M	Ulna	Yes	Yes	NR	None	12	Stable
					19-year-old/M	Humerus	Yes	Yes	Yes	None	11	Stable
					17-year-old/F	Tibia	Yes	Yes	Yes	None	17	Stable
					25-year-old/M	Spine	Yes	Yes	NR	Mild vomiting	23*	Stable
					19-year-old/M	Spine	Yes	Yes	NR	None	23*	Stable
[28]	Kurucu et al	2018	Turkey	9	16-year-old/M	Mandible	Yes	Yes	NR	NR**	20	Stable
					17-year-old/F	Pubic bone	Yes	Yes	NR	NR**	15	Recurrence
					12-year-old/M	Iliac bone	Yes	Yes	NR	NR**	10	Stable
					5-year-old/F	Spine	Yes	Yes	NR	NR**	12	Stable
					6-year-old/M	Acetabulum	Yes	Yes	Yes	NR**	20	Stable
					8-year-old/M	Humerus	Yes	No	NR	NR**	12	Stable
					16-year-old/F	Spine	Yes	Yes	No	NR**	21	Recurrence
					10-year-old/F	Spine	Yes	No	NR	NR**	24	Recurrence
					16-year-old/M	Sacrum	Yes	Yes	NR	NR**	12	Stable

Overall, there are indeed a limited number of studies that endeavored to investigate the role of denosumab in the management of patients with ABCs. Overall, the administration of denosumab appears to offer therapeutic benefits in terms of clinical (for example, symptom relief) and radiological (for example, tumor downsizing and ossification) responses in patients with ABCs. Furthermore, denosumab seems to be associated with safe drug profile, despite the sporadic occurrences of hypocalcemic events. The utilization of denosumab in ABCs is an off-label therapy and not yet approved by FDA. The employment of denosumab therapy in ABCs is largely justified by the histopathological closeness between GCTBs and ABCs [7,9-11]. Denosumab may emerge as a potential therapeutic agent in select patients with ABCs, particularly those patients presenting with locally advanced, recurrent, metastatic or inoperable diseases. Furthermore, denosumab my surface as a plausible therapy in ABC patients in whom the lesions are situated in anatomically challenging sites (for example, spine), or in ABC patients in whom initial surgical intervention is anticipated to result in substantial morbidity.

It should be clearly noted that the role of denosumab in ABCs cannot be concluded with certainty, owing to a multitude of limitations in the available literature. Such limitations include the weak study design in terms of sample size and study type. To elaborate further, the vast majority of studies were isolated single case reports, and even the retrospective cohort series included only a few patients (less than 10 patients) and such studies further lacked a valid control group. Moreover, to date, there are no randomized controlled trials which inform about highly trustworthy and strong evidence-based medicine. This can be largely attributed to that fact that the use of denosumab in the management of ABCs continues to be off-label and not yet green-lighted by the FDA. Additional limitations include variations in age of ABC populations treated with denosumab as well as the diverse dosing schedules of denosumab. With respect to survival and endpoint outcomes, the short duration of follow-up limits the prospect to withdraw concrete conclusions about short- and long-term efficacy, safety and loco-regional relapse. Lastly, the presence of diverse hidden confounding factors (for example, previous treatment with SAE) may have directly or indirectly influenced the true clinical/radiological outcomes in patients with ABCs.

## Conclusions

The use of RANKL-targeted therapy (denosumab) in the management of patients with ABCs is not FDA-approved and largely off-label. Taking into account the limited existing literature, denosumab therapy appears to offer therapeutic clinical and radiological benefits in select patients with ABCs, particularly those patients with locally advanced, recurrent or inoperable diseases. Large-sized randomized controlled trials are warranted in order to deduce solid conclusions about the efficacy and safety of denosumab in the management of a select cohort of patients with ABCs.
